# Changes in cannabis use according to socioeconomic status among Finnish adolescents from 2000 to 2015

**DOI:** 10.1186/s42238-020-00052-y

**Published:** 2020-12-24

**Authors:** Noora Knaappila, Mauri Marttunen, Sari Fröjd, Nina Lindberg, Riittakerttu Kaltiala

**Affiliations:** 1 Faculty of Medicine and Life Sciences, University of Tampere, 33014 Tampere, Finland; 2 Adolescent Psychiatry, University of Helsinki and Helsinki University Hospital, PO Box 22, FI-00014 Helsinki, Finland; 3 Department of Adolescent Psychiatry, Helsinki University Central Hospital, PO Box 590, 00029 HUS, Helsinki, Finland; 4 Vanha Vaasa Hospital, Vierinkiventie 1, 65380 Vaasa, Finland; 5 Department of Adolescent Psychiatry, Tampere University Hospital, 33380 Pitkäniemi, Finland

**Keywords:** Adolescent, Cannabis, Epidemiology, Socioeconomic factors, Socioeconomic status, Parental factors, Finland

## Abstract

**Abstract:**

**Background:**

Despite reduced sanctions and more permissive attitudes toward cannabis use in the USA and Europe, the prevalences of adolescent cannabis use have remained rather stable in the twenty-first century. However, whether trends in adolescent cannabis use differ between socioeconomic groups is not known. The aim of this study was to examine trends in cannabis use according to socioeconomic status among Finnish adolescents from 2000 to 2015.

**Methods:**

A population-based school survey was conducted biennially among 14–16-year-old Finns between 2000 and 2015 (*n* = 761,278). Distributions for any and frequent cannabis use over time according to socioeconomic adversities were calculated using crosstabs and chi-square test. Associations between any and frequent cannabis use, time, and socioeconomic adversities were studied using binomial logistic regression results shown by odds ratios with 95% confidence intervals.

**Results:**

At the overall level, the prevalences of lifetime and frequent cannabis use varied only slightly between 2000 and 2015. Cannabis use was associated with socioeconomic adversities (parental unemployment in the past year, low parental education, and not living with both parents). The differences in any and frequent cannabis use between socioeconomic groups increased significantly over the study period.

**Conclusions:**

Although the overall changes in the prevalence of adolescent cannabis use were modest, cannabis use increased markedly among adolescents with the most socioeconomic adversities. Socioeconomic adversities should be considered in the prevention of adolescent cannabis use.

## Background

In Finland, about 17% reported having used cannabis at some point in their lives (Varjonen 2012). The prevalence of cannabis use increased from the 1990s to the 2000s in the overall population, although it seems to have leveled off in the 2010s (Karjalainen et al. 2016; Varjonen 2012). Similarly, the attitudes toward cannabis use have become more permissive among Finns (Karjalainen et al. 2016). According to the ESPAD study conducted in 2015, eight percent of Finnish adolescents had tried cannabis in their lifetime (ESPAD Group 2016). As with cannabis use among adults, increase in adolescent cannabis use was rapid from the 1990s to the mid-2000s but slowed down after that (ESPAD Group 2016). The Finnish cannabis policy rests on total prohibition, which denotes that both use, possession, manufacturing, and selling are considered illegal (EMCDDA 2017), and the policy has not fundamentally changed in recent years (Varjonen 2012).

Despite being decriminalized in many countries, cannabis use has several detrimental effects on health that cannot be disregarded. The most comprehensive evidence exists on the association between cannabis use and mental health disorders, such as depression, anxiety, suicidal ideation, substance use, and psychosis among people with other genetic or environmental vulnerabilities (Chadwick et al. 2013; Karila et al. 2014; MacDonald and Pappas 2016; Patel et al. 2017; Volkow et al. 2014). Cannabis use also increases the risk for respiratory diseases and traffic accidents (Karila et al. 2014; MacDonald and Pappas 2016; Patel et al. 2017; Volkow et al. 2014). Furthermore, adolescent cannabis use is associated with social marginalization (Babor et al. 1978; Dugré et al. 2017; Varjonen 2012). In addition to individual suffering, drug-related harms cause substantial public expenditures, the largest parts in Finland going toward the enforcement of public order and safety, court and prison costs, and social services (Varjonen 2012).

Risk factors of cannabis use include easy availability (von Sydow et al. 2002), male gender (Gfroerer et al. 2007; ter Bogt et al. 2014; Thompson 2001), other substance use (Hayatbakhsh et al. 2009), peer and parental influences (Melchior et al. 2011; von Sydow et al. 2002), and positive attitudes toward cannabis (von Sydow et al. 2002). In addition, the association between cannabis use and socioeconomic status (SES) has been studied in the scientific literature. SES depicts an individual’s or group’s relative position within a society (Galobardes et al. 2006a; Galobardes et al. 2006b). It can be measured at both individual, household, and neighborhood levels (Krieger et al. 1997). It can be assessed not only through individual measures, such as education, income, or occupation (Galobardes et al. 2006a) but also through composite measures that provide an overall index of socioeconomic level. The measurement of adolescent SES is challenging as the final education and income levels are yet to be accomplished in adolescence. Therefore, parental SES is often used as a proxy measure of adolescent SES (Torikka et al. 2014; Torikka et al. 2017; Knaappila et al. 2018; Knaappila et al. 2019a; Knaappila et al. 2019b). Low socioeconomic status is a well-established risk factor of morbidity and mortality in all age groups (Clegg et al. 2009; Lund Jensen et al. 2017; Rawshani et al. 2016; Saydah et al. 2013). It affects health through several mechanisms, including living circumstances, lifestyle, and the consumption of health services (Øvrum 2011; Stringhini et al. 2011; Wang et al. 2015).

Of the socioeconomic factors, adolescent cannabis use has been associated with low parental education and not living with both parents (Delva et al. 2005; Henkel and Zemlin 2016; Swift et al. 2008; Widome et al. 2013). However, studies on the association between cannabis use and socioeconomic status are scarce. Substances tend to be more easily available in less affluent neighborhoods (Karriker-Jaffe 2013). According to Willis (1977), adolescents with low socioeconomic status may perceive certain behaviors that are common in low-SES groups as a reinforcement to their identities. Therefore, adolescents from low socioeconomic backgrounds may view substance use, that is more common in low-SES groups than in other socioeconomic groups, as something to pursue in an effort to reinforce their identity.

Scientific evidence suggests that recent decades have seen an increase in socioeconomic health disparities in the Nordic countries, including Finland. Matthiessen et al. (2014) observed that the prevalence of overweight increased in Denmark between 2000 and 2008 in boys of parents with low educational level only. Similarly, socioeconomic disparities increased in self-rated health among Swedish adolescents between 2002 and 2014 (Ahlborg et al. 2017). Torikka et al. (2017, 2014) found that the differences in the prevalences of depression, frequent drinking, and drunkenness between socioeconomic groups increased among Finnish adolescents from 2000 to 2011. Therefore, although changes in the general prevalence of cannabis use have been modest in the twenty-first century, there may be differing trends between socioeconomic groups. However, studies investigating socioeconomic trends in adolescent cannabis use have not yet been conducted.

## Methods

The aim of this study was to examine time-trends in the prevalence of cannabis use according to socioeconomic status among Finnish adolescents. Data for the study was obtained from The School Health Promotion Study of the National Institute for Health and Welfare, which is a survey on the health, health behavior, and school experiences of Finnish adolescents. The survey has been conducted biennially since 1996 among Finnish 8th and 9th graders with pooled 2-year data. In this study, we used data collected between 2000 and 2015. The data were collected anonymously during a school lesson under the supervision of a teacher, who did not interfere with the responses. Participants were informed orally and in writing about the voluntary nature of the study, and returning the survey was considered consent to participate. The survey was done by paper and pencil and it took about 30–45 min to complete. After this, the surveys were put in an envelope, sealed, and returned directly to the research center. The timing of the study, sampling, and data collection methods were held constant in each survey. Altogether, 761,278 (50,404–109,127 biennially) 8th and 9th graders participated in the survey. 8th graders were 14-15 years old and 9th graders 15–16 years old at the time of the surveys. The biennial cohorts covered between 74 and 84% of the whole age cohort of the country. The study was approved by the ethics committee of Pirkanmaa Hospital District and the National Institute of Health and Welfare.

Lifetime substance use was elicited as follows: “Have you ever tried or used the following substances? Please answer every item.” The response alternative measuring lifetime cannabis use was “marijuana or hashish.” The formulation of the question was held constant across all questionnaires, except that in the 2015 questionnaire the response alternative was slightly more specified: “marijuana or hashish (cannabis).” The response alternatives were “never/once/2–4 times/5 times or more often.” For the analyses, two dichotomous variables—“any cannabis use” (at least once vs never) and “frequent cannabis use” (5 times or more often vs other)—were created. The cut-off point of five or more times was chosen as Zoccolillo et al. (1999) demonstrated that such frequency indicates problematic use in this age group.

The socioeconomic variables recorded were parental education, parental unemployment in the past year, and family structure. The associations between all three variables and adolescent health are well established (Areba et al. 2018; Sleskova et al. 2006; Torikka et al. 2017). Parental education was elicited as follows: “What is the highest educational qualification your father/mother has achieved?” The response options in the 2000 questionnaire were “basic school/vocational school/high school and/or vocational school/university or polytechnic.” The response options varied a little over time: for instance, in the 2013 questionnaire there was a response option “no education,” which was removed again in the 2015 questionnaire. For the analyses, parental education was dichotomized as parental basic education only (including the response alternatives “basic school” and “no education”) versus other. Parental unemployment was elicited as follows: “Have your parents been unemployed or laid off work during the past YEAR?” The response alternatives were the same in all questionnaires: “neither/one parent/both parents.” The family structure was elicited as follows: “My family consists of...” The response options in the 2000 questionnaire were “mother and father/mother and stepfather/father and stepmother/mother only/father only/spouse/other caregiver.” The response options varied slightly over time. For the analyses, family structure was dichotomized as living with both parents versus other. In this article, all three variables are referred to as socioeconomic adversities. In addition, a variable “cumulative socioeconomic adversity” was created, in which all three socioeconomic variables were combined: a score of 0 stood for having no socioeconomic adversities (living with both parents, no parental unemployment, at least one parent with higher than basic education) and a score of 4 stood for having all socioeconomic adversities (not living with both parents, both parents unemployed, both parents with basic education only). The prevalences of socioeconomic adversities over time are presented elsewhere (Knaappila et al. 2018).

The distributions of any and frequent cannabis use across years among boys and girls in the whole sample as well as according to cumulative socioeconomic adversity were studied using crosstabulation and chi-square test. Less than 3% of the responses were missing in all variables apart from parental education, which had missing values of 7.6% in boys and 6.6% in girls. As all the results were statistically significant (*p* < 0.01), the *p* values were not addressed in the tables. Bivariate associations were studied using binomial logistic regression results shown by odds ratios with 95% confidence intervals. Any and frequent cannabis use were entered as dependent variables. In the first model, categorical time periods (2000–2001, 2002–2003, 2004–2005, 2006–2007, 2008–2009, 2010–2011, 2012–2013, 2014–2015) were entered as an independent factor using the time period 2000–2001 as a reference category. In the second model, family structure (living with both parents/other), parental unemployment in the past year (neither/one parent/both parents), and parental education (both parents basic education only/other) were entered as an independent factor one at a time. In the third model, the file was split according to categorical time periods and cumulative socioeconomic adversity was entered as an independent factor.

## Results

Of girls 6% and of boys 8% reported having used cannabis at least once in their lifetime. Of girls 2% and of boys 3% reported having used cannabis five times or more often (Table 1). Both any and frequent cannabis use varied only slightly between years among both sexes (Table 2).

**Table 1:** Distribution of any and frequent cannabis use and socioeconomic adversities among boys and girls in the 8th or 9th grades of comprehensive school in Finland between 2000 and 2015 (%)

	Boys (*n* = 381527)	Girls (*n* = 376814)
Age, years (mean (sd))	15.4 (0.7)	15.3 (0.6)
Lifetime cannabis use^a^		
Yes	8.1	6.0
No	90.7	93.3
Frequent cannabis use^b^		
Yes	2.9	1.5
No	95.9	97.9
Lives with both parents		
Yes	74.4	73.7
No	23.3	25.1
Both parents only basic education		
Yes	5.6	5.9
No	86.8	87.5
Parental unemployment past year		
No	70.9	69.9
One parent	23.6	25.6
Both parents	3.2	3.3

^a^Used cannabis at least once in one’s lifetime

^b^Used cannabis at least five times in one’s lifetime

**Table 2:** Distribution of any^a^ and frequent^b^ cannabis use across years among boys and girls in the 8th or 9th grades of comprehensive school in Finland (%)

Boys								
	2000–2001 (*n* = 42685)	2002–2003 (*n* = 51309)	2004–2005 (*n* = 53499)	2006–2007 (*n* = 54841)	2008–2009 (*n* = 54433)	2010–2011 (*n* = 51329)	2012–2013 (*n* = 50223)	2014–2015 (*n* = 25147)
Any cannabis use	9.1	9.1	7.1	6.1	6.5	8.6	9.8	10.1
Frequent cannabis use	2.9	3.0	2.4	2.1	2.2	3.2	3.7	5.0
Girls								
	2000–2001 (*n* = 42122)	2002–2003 (*n* = 49481)	2004–2005 (*n* = 51979)	2006–2007 (*n* = 54286)	2008–2009 (*n* = 54216)	2010–2011 (*n* = 51216)	2012–2013 (*n* = 49255)	2014–2015 (*n* = 25257)
Any cannabis use	8.0	7.7	6.0	4.6	4.4	5.8	6.4	6.2
Frequent cannabis use	1.8	1.7	1.3	1.0	1.0	1.3	1.7	2.2

^a^Used cannabis at least once in one’s lifetime

^b^Used cannabis at least five times in one’s lifetime

The associations between cannabis use and socioeconomic adversities are presented in Table 3. Any and frequent cannabis use were more common among girls and boys who did not live with both parents than among those who lived with both parents. Cannabis use was positively associated with parental unemployment: any and frequent cannabis use were most common among girls and boys whose both parents had been unemployed and least common among those whose parents had not been unemployed in the past year. Any and frequent cannabis use were also more common when both parents had only basic education than when at least one parent had higher than basic education. Socioeconomic differences were greater in frequent cannabis use than any cannabis use.

**Table 3:** Odds ratios (95% confidence intervals) for any^a^ and frequent^b^ cannabis use according to socioeconomic adversities among Finnish boys and girls in the 8th and 9th grades of comprehensive school in 2000-2015

	Boys		Girls	
	Any cannabis use	Frequent cannabis use	Any cannabis use	Frequent cannnabis use
Family structure				
Living with both parents	Ref	Ref	Ref	Ref
Not living with both parents	2.7 (2.6–2.7)	3.1 (3.0–3.3)	2.8 (2.7–2.8)	3.7 (3.5–3.9)
Both parents with low education				
No	Ref	Ref	Ref	Ref
Yes	1.9 (1.8–2.0)	3.2 (3.1–3.4)	1.5 (1.4–1.5)	2.6 (2.4–2.8)
Parental unemployment				
Neither parent	Ref	Ref	Ref	Ref
One parent	1.5 (1.4–1.5)	1.4 (1.3–1.5)	1.6 (1.5–1.6)	1.7 (1.6–1.8)
Both parents	4.6 (4.4–4.8)	8.4 (7.9–8.8)	3.1 (2.9–3.2)	6.1 (5.6–6.6)

^a^Used cannabis at least once in one’s lifetime

^b^Used cannabis at least five times in one’s lifetime

The more socioeconomic adversities accumulated, the more common any and frequent cannabis use were. Prevalences in any and frequent cannabis use increased markedly among adolescents with most cumulative socioeconomic adversity over time (Table 4). Differences in any cannabis use between girls not living with both parents, with both parents unemployed and parental basic education only and girls living with both parents, with no parental unemployment, and at least one parent with higher than basic education increased from 2000–2001 (OR = 4.6, 95% CI 2.8–7.7) to 2014–2015 (OR = 36.6, 95% CI 23.9–55.9). Similarly, differences in any cannabis use between socioeconomic groups increased among boys from 2000–2001 (OR = 12.0, 95% CI 8.2–17.6) to 2014–2015 (OR = 51.4, 95% 36.6–72.0). Socioeconomic differences in frequent cannabis use among girls increased from 2000–2001 (OR = 7.9, 95% CI 3.6–17.4) to 2014–2015 (OR = 101.9, 95% CI 64.1–162.3). Likewise, socioeconomic differences in frequent cannabis use increased among boys from 2000–2001 (OR = 29.5, 95% CI 19.7–44.2) to 2014–2015 (OR = 101.9, 95% CI 64.1–162.3) (Table 5).

**Table 4:** Distributions of any^a^ and frequent^b^ cannabis use according to socioeconomic adversities^c^ across years among boys and girls in the 8th or 9th grades of comprehensive school in Finland (%)

	2000–2001	2002–2003	2004–2005	2006–2007	2008–2009	2010–2011	2012–2013	2014–2015
Boys, any cannabis use								
Number of socioeconomic adversities								
0	6.8	6.5	4.9	3.9	4.5	5.9	6.2	5.7
1	9.5	10.1	8.0	6.7	7.2	9.2	10.2	10.2
2	15.4	16.7	12.0	12.3	11.6	15.1	15.9	15.1
3	20.8	24.9	24.0	25.5	27.1	26.2	26.7	27.3
4	46.8	58.9	58.5	65.6	71.6	71.4	61.1	75.8
Girls, any cannabis use								
Number of socioeconomic adversities								
0	5.9	5.6	3.9	2.9	2.8	3.7	3.6	3.0
1	8.3	8.5	6.9	5.6	4.9	6.2	6.9	6.6
2	12.7	12.8	10.9	9.6	8.5	10.6	11.0	11.1
3	14.3	17.3	15.1	15.0	13.9	13.1	14.1	17.8
4	22.4	32.6	44.9	39.2	48.0	47.5	43.9	53.3
Boys, frequent cannabis use								
Number of socioeconomic adversities								
0	1.9	1.9	1.5	1.1	1.3	1.9	1.9	2.2
1	2.7	3.0	2.3	2.0	2.1	3.1	3.4	4.6
2	5.2	5.9	3.8	4.5	4.0	5.7	5.9	7.1
3	10.6	12.5	13.1	15.0	15.9	14.9	14.4	14.4
4	35.8	44.2	44.9	54.2	63.1	59.4	54.3	71.1
Girls, frequent cannabis use								
Number of socioeconomic adversities								
0	1.1	1.0	0.6	0.5	0.6	0.6	0.7	0.7
1	2.0	1.7	1.5	1.2	1.0	1.3	1.6	2.2
2	3.2	3.2	2.4	2.4	1.8	2.4	3.3	4.2
3	4.0	6.1	5.3	5.9	5.4	4.9	5.6	7.5
4	8.2	16.3	33.7	27.8	38.2	36.4	31.0	43.5

^a^Used cannabis at least once in one’s lifetime

^b^Used cannabis at least five times in one’s lifetime

^c^Socioeconomic adversities included not living with both parents, both parents with basic education only, and both parents unemployed

**Table 5:** Odds ratios (95% confidence intervals) for any^a^ and frequent^b^ cannabis use according to cumulative number of sociodemographic adversities^c^ in the different study years, among boys and girls in the 8th or 9th grades of comprehensive school in Finland

	2000–2001	2002–2003	2004–2005	2006–2007	2008–2009	2010–2011	2012–2013	2014–2015
Boys, any cannabis use								
Number of adversities								
0	Ref	Ref	Ref	Ref	Ref	Ref	Ref	Ref
1	1.4 (1.3–1.6)	1.6 (1.5–1.7)	1.7 (1.6–1.8)	1.8 (1.6–1.9)	1.7 (1.5–1.8)	1.6 (1.5–1.8)	1.7 (1.6–1.9)	1.9 (1.7–2.1)
2	2.5 (2.3–2.7)	2.9 (2.7–3.2)	2.7 (2.4–3.0)	3.4 (3.1–3.8)	2.8 (2.5–3.1)	2.9 (2.6–3.1)	2.9 (2.6–3.2)	2.9 (2.6–3.3)
3	3.6 (3.0–4.3)	4.8 (4.1–5.7)	6.2 (5.2–7.3)	8.4 (7.0–10.0)	8.0 (6.7–9.5)	5.7 (4.9–6.7)	5.6 (4.8–6.5)	6.2 (5.0–7.6)
4	12.0 (8.2–17.6)	20.7 (14.5–29.5)	27.6 (19.0–39.9)	46.6 (32.4–67.2)	54.2 (37.4–78.4)	40.2 (28.9–56.0)	23.9 (18.2–31.5)	51.4 (36.6–72.0)
Girls, any cannabis use								
Number of adversities								
0	Ref	Ref	Ref	Ref	Ref	Ref	Ref	Ref
1	1.5 (1.3–1.6)	1.6 (1.4–1.7)	1.9 (1.7–2.0)	2.0 (1.8–2.2)	1.8 (1.6–1.9)	1.7 (1.6–1.9)	2.0 (1.8–2.2)	2.3 (2.0–2.6)
2	2.3 (2.1–2.6)	2.5 (2.2–2.7)	3.0 (2.7–3.4)	3.6 (3.2–4.0)	3.2 (2.8–3.6)	3.1 (2.8–3.4)	3.3 (3.0–3.7)	4.0 (3.4–4.6)
3	2.7 (2.2–3.2)	3.5 (2.9–4.2)	4.4 (3.6–5.4)	6.0 (4.9–7.4)	5.5 (4.5–6.9)	3.9 (3.2–4.7)	4.4 (3.7–5.3)	6.9 (5.5–8.7)
4	4.6 (2.8–7.7)	8.1 (5.2–12.6)	20.4 (13.4–31.1)	21.8 (14.5–33.0)	31.6 (21.3–46.9)	23.3 (17.0–32.0)	21.0 (15.4–28.6)	36.6 (23.9–55.9)
Boys, frequent cannabis use								
Number of adversities								
0	Ref	Ref	Ref	Ref	Ref	Ref	Ref	Ref
1	1.5 (1.3–1.7)	1.7 (1.5–1.9)	1.6 (1.4–1.8)	1.8 (1.6–2.1)	1.6 (1.4–1.9)	1.6 (1.4–1.8)	1.9 (1.6–2.1)	2.1 (1.8–2.5)
2	2.9 (2.5–3.4)	3.3 (2.8–3.8)	2.7 (2.2–3.2)	4.2 (3.6–5.1)	3.2 (2.7–3.9)	3.1 (2.7–3.6)	3.3 (2.9–3.9)	3.3 (2.8–4.0)
3	6.3 (5.0–8.0)	7.6 (6.1–9.4)	10.0 (8.0–12.5)	15.6 (12.4–19.7)	14.5 (11.5–18.3)	8.9 (7.3–11.0)	8.9 (7.2–11.0)	7.3 (5.6–9.6)
4	29.5 (19.7–44.2)	41.8 (29.2–59.8)	54.3 (37.3–79.1)	105.3 (73.5–150.9)	131.7 (92.2–188.2)	74.5 (54.4–102.0)	62.9 (47.5–83.4)	107.3 (76.8–150.0)
Girls, frequent cannabis use								
Number of adversities								
0	Ref	Ref	Ref	Ref	Ref	Ref	Ref	Ref
1	1.8 (1.5–2.1)	1.6 (1.3–1.9)	2.5 (2.0–3.1)	2.4 (1.9–3.0)	1.8 (1.5–2.3)	2.0 (1.7–2.5)	2.5 (2.0–3.0)	3.0 (2.3–3.9)
2	2.9 (2.4–3.6)	3.2 (2.6–3.9)	4.0 (3.2–5.0)	5.0 (3.9–6.3)	3.4 (2.6–4.4)	3.9 (3.1–4.9)	5.0 (4.0–6.2)	5.8 (4.5–7.6)
3	3.6 (2.6–5.2)	6.2 (4.6–8.3)	9.0 (6.4–12.5)	12.5 (8.9–17.7)	10.3 (7.2–14.6)	8.1 (5.9–11.1)	8.8 (6.5–12.0)	10.8 (7.5–15.4)
4	7.9 (3.6–17.4)	18.6 (10.5–32.7)	81.0 (50.9–129.0)	77.1 (48.1–123.7)	111.6 (72.7–171.5)	89.1 (62.4–127.1)	66.4 (46.2–95.4)	101.9 (64.1–162.3)

^a^Used cannabis at least once in one’s lifetime

^b^Used cannabis at least five times in one’s lifetime

^c^Socioeconomic adversities included not living with both parents, both parents with basic education only, and both parents unemployed. Adolescents living with both parents, with at least one parent with higher than basic education and both parents employed used as a reference group

## Discussion

In this study, we found that cannabis use was associated with socioeconomic adversities among Finnish adolescents. The prevalences of any and frequent cannabis use were higher among adolescents not living with two parents than among those living with both parents. Similarly, the prevalences of any and frequent cannabis use were higher among adolescents with parental unemployment in the past year and adolescents with parental basic education only than among adolescents without these socioeconomic adversities. The more socioeconomic adversities an adolescent had, the more likely they used cannabis. The most important, and novel, finding was that cannabis use increased markedly from 2000 to 2015 among adolescents with most socioeconomic adversities.

The association between cannabis use and low parental education, as well as that between adolescent cannabis use and not living with both parents, have been observed in earlier studies (Delva et al. 2005; Henkel and Zemlin 2016; Swift et al. 2008; Widome et al. 2013). To our knowledge, no previous studies have investigated the association between adolescent cannabis use and parental unemployment. There are many possible explanations for these associations. First, all the socioeconomic adversities studied are associated with lower income level in the family, which in turn is a risk factor of adolescent cannabis use (Tu et al. 2008; von Sydow et al. 2002). Parents with higher education level may have more knowledge on the harms of cannabis and therefore more negative attitudes toward cannabis use (Wu et al. 2015). On the other hand, adults with low education are more likely to use substances (Weitoft et al. 2008), and parental substance use is a major risk factor of adolescent substance use (Hoffmann and Cerbone 2002). The prevalence of mental health problems, which are associated with cannabis use, is also higher among children not living with both parents and those with parental unemployment (Kedzior and Laeber 2014; Pedersen et al. 2001). The associations were stronger for frequent cannabis use than lifetime cannabis use, which is in accordance with the knowledge that socioeconomic adversities are more strongly associated with heavier substance use (Lund 2015; Torikka et al. 2017).

Changes in the prevalence of both lifetime and frequent cannabis use were modest in the overall population. This finding is in accordance with earlier studies (Arnarsson et al. 2018; Delva et al. 2005; Gfroerer et al. 2007; Henkel and Zemlin 2016; Johnson et al. 2015; ESPAD Group 2016; ter Bogt et al. 2014). However, among adolescents with most socioeconomic adversities, the likelihood of both lifetime and frequent cannabis use increased significantly over the study period. The finding is surprising as time trends in adolescent cannabis use according to the socioeconomic status have not been studied previously. One explanation to the findings might be liberalized attitudes toward cannabis use in Finland (Karjalainen et al. 2016). Wardle and Steptoe (2003) observed in their study that unhealthy attitudes were associated with low socioeconomic status. Attitudinal changes may have affected the increase in cannabis use among adolescents with most socioeconomic adversities. In addition, increased disparities in substance use among adults might explain the widening disparities in cannabis use among adolescents (Rotko et al. 2011). Also, availability of cannabis has increased in Finland due to anonymous online markets (Nurmi et al. 2017), which may have increased cannabis use among those most prone to use it. The increasing engagement of youth in the online world may have contributed differently to the behaviors of adolescents in different socioeconomic groups.

Cannabis use is strongly correlated with smoking, and recently increased socioeconomic disparities have also been observed in adolescent smoking (Knaappila et al. 2019a). Increased disparities in smoking may therefore have enhanced disparities in cannabis use. Increased socioeconomic health disparities have also been observed in other areas of adolescent health in Finland and other countries (Knaappila et al. 2018; Knaappila et al. 2019b; Matthiessen et al. 2014; Moraeus et al. 2014; Torikka et al. 2014; Torikka et al. 2017). Torikka et al. (2017, 2014) observed increased socioeconomic differences in depression and alcohol consumption among Finnish adolescents between 2000 and 2011. Similarly, Knaappila et al. (2018, 2019b) observed that the prevalences of delinquency and bullying at school increased among adolescents with low socioeconomic status only. In Finland, the economic depression in the 1990s led to marked increases in unemployment (Rotko et al. 2011). Also, the purchasing power of people on social security benefits has decreased in the past decades (Rotko et al. 2011). These societal changes may have contributed to increases in socioeconomic health disparities in Finland. It can also be that the identity processes of adolescents from different socioeconomic backgrounds are differing in a way that has led to increased socioeconomic disparities in health behaviors among adolescents (Willis 1977). As the socioeconomic disparities are rooted in societal structures, socioeconomic health disparities can be decreased through socio-political decision-making. Securing everyone’s equal access to education, work, and social and health services are important ways to decrease socioeconomic disparities in health and well-being, including cannabis use. In addition, socioeconomic adversities should be considered in the prevention of cannabis use among adolescents.

This study has some limitations. Self-report data is susceptible to errors, such as recall bias and invalid responding. Parental education especially may be difficult for an adolescent to recall, which may have caused the proportion of missing responses to be higher on that question than other questions. However, the proportions of missing responses on all questions studied were small and fluctuated slightly without displaying any clear trend that could influence the trends of interest. Invalid responding is another source of error in studies relying on self-report data. Social desirability may result in too low reporting of problem behaviors (Fisher and Katz 2008), and adolescents may also find it funny to exaggerate their symptoms and problem behaviors in questionnaires (Robinson-Cimpian 2014). Such influences on cannabis use were not controlled for, but there is no reason to assume that either social desirability or exaggerating problems biased the trends identified.

Despite the limitations, this study has several strengths: it is based on a unique nationwide time trend data with a large sample size consisting of Finnish 8th and 9th graders (*n* = 761,278). The biennial cohorts covered between 74 and 84% of the whole age cohort of the country. The sampling and timing of the study as well as the formulation of questions were held constant over the years. Instead of one measure of cannabis use, we were able to study both any and frequent use. In addition, several measures were used to assess socioeconomic adversity.

## Conclusion

This study found that socioeconomic disparities in cannabis use increased among Finnish adolescents between 2000 and 2015. Although changes in the overall prevalence of cannabis use were modest, cannabis use increased among adolescents with most socioeconomic adversities. Socioeconomic health disparities increase individual suffering and inflict a great burden on public health and economy (Koskinen and Martelin 2007). Securing everyone’s equal access to education, work, and social and health services are important ways to decrease socioeconomic disparities in health and well-being, including cannabis use. In addition, socioeconomic adversities should be considered in the prevention of cannabis use among adolescents.
